# The Role of Sumoylation in the Response to Hypoxia: An Overview

**DOI:** 10.3390/cells9112359

**Published:** 2020-10-26

**Authors:** Chrysa Filippopoulou, George Simos, Georgia Chachami

**Affiliations:** 1 Laboratory of Biochemistry, Faculty of Medicine, University of Thessaly, 41500 Larissa, Greece; cfilippop@uth.gr (C.F.); simos@med.uth.gr (G.S.); 2 Gerald Bronfman Department of Oncology, Faculty of Medicine, McGill University, Montreal, QC H4A 3T2, Canada

**Keywords:** hypoxia, HIF, HIF-1α, oxygen homeostasis, SUMO, sumoylation

## Abstract

Sumoylation is the covalent attachment of the small ubiquitin-related modifier (SUMO) to a vast variety of proteins in order to modulate their function. Sumoylation has emerged as an important modification with a regulatory role in the cellular response to different types of stress including osmotic, hypoxic and oxidative stress. Hypoxia can occur under physiological or pathological conditions, such as ischemia and cancer, as a result of an oxygen imbalance caused by low supply and/or increased consumption. The hypoxia inducible factors (HIFs), and the proteins that regulate their fate, are critical molecular mediators of the response to hypoxia and modulate procedures such as glucose and lipid metabolism, angiogenesis, erythropoiesis and, in the case of cancer, tumor progression and metastasis. Here, we provide an overview of the sumoylation-dependent mechanisms that are activated under hypoxia and the way they influence key players of the hypoxic response pathway. As hypoxia is a hallmark of many diseases, understanding the interrelated connections between the SUMO and the hypoxic signaling pathways can open the way for future molecular therapeutic interventions.

## 1. Introduction

Post-translational modifications are critical events in cellular signaling that allow regulation and fine-tuning of protein function. Sumoylation is a post-translational modification that involves the covalent attachment of the 97-amino acid SUMO (small ubiquitin-like modifier) protein to lysine residues of target proteins [1,2,3]. SUMO was originally discovered by its ability to modify RanGAP1. Sumoylation of RanGAP1 is required for its localization at the nuclear pore complexes and its interaction with RanBP2, which is essential for the role of RanGAP1 in the protein nuclear import [4,5]. It has been subsequently shown that modification by SUMO is implicated in the nucleocytoplasmic shuttling of many proteins [6]. However, studies over the years revealed that SUMO targets not only control nuclear transport mechanisms but rather participate in nearly all aspects of cell biology and sumoylation has been established as one of the most common reversible protein modifications implicated in cellular processes ranging from signal transduction and ion transport to transcription, recombination and chromosome segregation and DNA repair [7,8]. Protein sumoylation is highly dynamic, enabling rapid responses to external and internal stimuli [9]. Many studies have shown the involvement of sumoylation in the response to different kinds of cellular stress [10,11,12]. In this review, we will focus on the SUMO modifications that are implicated in the cellular adaptation to hypoxia and will highlight the significance of sumoylation targeting factors of the hypoxia signaling pathway.

## 2. The SUMO Proteins

The SUMO proteins belong to the family of ubiquitin-like proteins (Ubls), which are characterized by the presence of the ubiquitin or β-grasp fold, a structure of four-stranded β-sheet wrapped around a central α-helix [13]. Most Ubls, including SUMOs, contain a glycine-glycine motif at their C-terminal end, which, when exposed by proteolysis, is responsible for the conjugation to their target protein. One feature that sets SUMO proteins apart from the rest of the Ubl family is their 20 amino acid long N-terminal end. SUMO2/3 contain consensus sumoylation sites in their N-terminal extension, which are critical for the attachment of SUMOs and the formation of SUMO chains [14]. However, the role of polysumoylation is still under investigation.

SUMO proteins are conserved throughout eukaryotes. Higher eukaryotes such as vertebrates contain multiple SUMO genes. In humans, distinct SUMO genes have been found to code for different SUMO proteins, SUMO1-5. Mature SUMO2 and SUMO3 consist of 92 and 93 amino acids, respectively, and show 97% similarity. Due to this similarity, they are often referred to as a single SUMO2/3 protein family and are indistinguishable by specific antibodies. Due to inconsistencies on the used nomenclature for SUMO2 and SUMO3, herein we used the nomenclature as described by Saitoh and Hinchey [15]. SUMO1 presents 47% similarity to SUMO2/3 [16]. SUMO1–3 proteins are expressed ubiquitously, with the SUMO2/3 pool being comparatively bigger than SUMO1 [15]. SUMO4 and SUMO5 are much less investigated and, if expressed at all, they demonstrate tissue-specific expression [17,18,19,20].

## 3. The SUMO Conjugation Pathway

**Figure 1:**
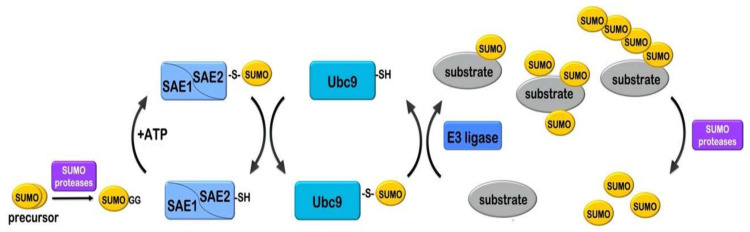
The SUMO conjugation mechanism. Initially the SUMO precursor form is proteolytically cleaved (by SUMO-specific proteases) to expose the C-terminal glycine–glycine (GG) motif. Mature SUMO is activated by the SUMO E1 heterodimer SAE1/SAE2 (Aos1/Uba2) in an ATP-dependent manner, then a thioester bond is formed between the C-terminal glycine of SUMO and the catalytic cysteine (-SH) of SAE2. SUMO is subsequently loaded to the catalytic cysteine of the SUMO E2 enzyme Ubc9. Ubc9 catalyzes formation of an isopeptide bond between the C-terminal glycine of SUMO and a lysine residue in the substrate. This procedure is usually facilitated by a SUMO E3 ligase. Sumoylation can occur as mono-sumoylation, poly-mono-sumoylation or polysumoylation (formation of sumo chains) on target substrates. Sumoylation is reversed by SUMO-specific proteases that cleave the isopeptide bond and release SUMO.

Conjugation of SUMOs with target proteins is facilitated by SUMO-specific enzymes (E1, E2 and E3), resembling the enzymatic mechanism of Ubl conjugation (Figure 1). Initially, SUMO proteins, which are expressed as precursor forms, are subjected to “maturation”, in order to reveal their glycine–glycine motif [21]. This process is enabled by a category of SUMO specific proteases. Mature SUMO proteins are activated by the SUMO-specific E1 enzyme, using energy released form ATP hydrolysis. The E1 enzyme is a heterodimer consisting of 2 subunits, namely SUMO-activating enzyme subunit 1 and 2 (SAE1/SAE2, also known as Aos1/Uba2) [22,23,24]. The SAE1/SAE2 heterodimer uses importin α/β in order to enter the nucleus [25]. SAE1/SAE2 displays two adenylation domains, a catalytic cysteine domain and a carboxy-terminal ubiquitin-fold domain (UFD domain) [13,26,27,28]. Initially, the E1 heterodimer recognizes SUMO and ATP-Mg via its adenylation sites and initiates the conjugation process by performing adenylation of the SUMO C-terminus. Subsequently, a thioester bond forms between SUMO and the active-site cysteine residue of E1 [29]. Finally, the UFD domain of E1 activating enzyme interacts with the E2 conjugating enzyme Ubc9 and the activated SUMO is transferred to a new cysteine in the catalytic center of E2 [30,31]. Unlike the ubiquitin pathway that contains many different E2 enzymes, Ubc9 is the only known conjugating enzyme for all SUMOs. Ubc9 has the ability to select and bind directly to target proteins by identifying a specific ψKxD/E motif, where ψ corresponds to an aliphatic branched amino acid residue, K is the lysine to be modified, followed by any amino acid and an aspartate or glutamate residue. Although, half of the SUMO targets have been found to contain the typical SUMO consensus motif, there are many cases of protein substrates in which sumoylation occurs on lysine residues of alternative motifs [1,26,32]. In sοme cases, the substrate also contains a SUMO-interacting motif (SIM) that recruits SUMO, near lysine residues outside of a consensus motif [33,34].

Despite the ability of Ubc9 to recognize and bind to target proteins, several cases require cooperation of Ubc9 with a specific SUMO E3 ligase in order to achieve efficient coupling with the target. SUMO E3 enzymes catalyze the discharging of SUMO from Ubc9 and its transfer to the lysine residue of the target protein. So far, several proteins have been characterized as SUMO E3 ligases mostly due to their ability to enhance sumoylation of targets like the PIAS family, Topors, RanBP2, Pc2, p14Arf, Krox20, SF2/ASF and more [35,36,37,38,39,40,41]. Biochemical and structural data confirmed the E3 ligase function of the PIAS family [35], RanBP2/Nup358 [37,42] and ZNF451 [43]. 

The highly dynamic and reversible nature of the SUMO modification is based on the action of SUMO specific proteases. SUMO proteases belong to the cysteine protease family and they are responsible for the cleavage of the isopeptide bond that links SUMO to the ε-amino group of the lysine of the target protein (deconjugation reaction) or the cleavage of SUMO from poly-SUMO2/3 chains [44,45]. Finally, as already discussed above, SUMO specific proteases are also responsible for the processing of SUMO, hydrolyzing a peptide bond close to the C-terminus of SUMO precursor, and exposing two glycine residues (the diGly motif), which is a prerequisite for SUMO activation and conjugation [21].

The known SUMO proteases are classified into three distinct families: the Ulp/SENP (ubiquitin-like protease/sentrin-specific protease) family, the Desi (deSUMOylating isopeptidase) family [46] and USPL1 (ubiquitin-specific peptidase-like protein 1) [47]. In humans, seven members of the SENP family have been discovered, so far, SENP1, 2, 3, 5, 6, 7 and SENP8 or DEN1 [45,48,49,50]. These SENPs can be divided into three subfamilies based on their sequence homology, substrate specificity and subcellular localization, the SENP1 and SENP2 category, the SENP3 and SENP5 and, finally, the SENP6 and SENP7 [51]. DEN1 is not considered a SUMO specific enzyme as it acts on NEDD8 and not SUMO [50]. SENP1 and SENP2 participate in both processing and deconjugation reactions without any particular preference in any SUMO isoform [52]. They are both mostly localized in the nucleus and especially the nuclear pore complex [53]. However, SENP2 isoforms have been also detected in PML nuclear bodies and the cytosol [54]. SENP3 and SENP5 are localized in the nucleolus via binding to NPM1 [55] and they exhibit a pronounced preference for SUMO2/3 substrates [52]. Finally, SENP6 and SENP7 are mostly nuclear. Although they are considered inactive in the SUMO maturation process, they are the most efficient SENP isoforms in cleavage of di- and poly-SUMO2/3 chains [56]. The Desi family consists of deSUMOylating isopeptidases Desi-1 and Desi-2. BZEL, a transcription corepressor is the only known substrate of Desi-1 so far [46].

The most recently identified SUMO isopeptidase is the ubiquitin-specific protease-like 1 (USPL1), which plays an essential role in Cajal body biology [47,57].

## 4. Sumoylation under Stress

Sumoylation states are highly dynamic, enabling rapid responses to external and internal stimuli [9]. A rapidly expanding field of study is focusing on investigating the involvement of sumoylation in the response to stress conditions [10,12]. Adaptive response mechanisms may include regulation of the sumoylation of a specific target protein (e.g., by altering its phosphorylation state, which in turn affects its sumoylation) or fine-tuning of the SUMO conjugation machinery in order to alter global sumoylation levels.

The sumoylation pathway has been extensively studied under stress conditions such as heat shock, oxidative stress, ischemia (see below), hypothermia and viral infection in mammals but also under genotoxic stress and osmotic stress in yeast and plants [34,58,59,60,61]. Upon heat shock, a rapid accumulation of SUMO2/3 modifications was observed in a wide range of substrates [59,62,63,64]. The majority of these substrates are nuclear proteins and especially chromatin interacting proteins. It is suggested that these modifications aim at the regulation of transcription via modulation of Pol II pausing, which might be important for the maintenance and organization of chromatin structure [65,66]. Recently, the increase of heat shock mediated SUMO2/3 conjugation was proposed to coordinate proteome degradation and assist the maintenance of proteostasis upon proteotoxic stress [67]. On the contrary, oxidative stress leads to the blockage of the sumoylation machinery with consequent reduction of global sumoylation of proteins. Specifically, exposure to H_2_O_2_ (around 1 mM) leads to oxidation of E1 and E2 enzymes, formation of a disulfide bridge between their catalytic cysteines and consequent inactivation of both enzymes [58,68]. This inactivation is considered beneficial for the cell by promoting survival during the oxidative DNA damage response [68]. Moreover, other components of the catalytic machinery like SENPs 1, 2 and 3 are also known targets of H_2_O_2_ [69,70] (for review see [11]). Our review focuses on the involvement of SUMO modifications in the cellular response to hypoxia.

## 5. Hypoxia and the Hypoxic Signaling Cascade

**Figure 2:**
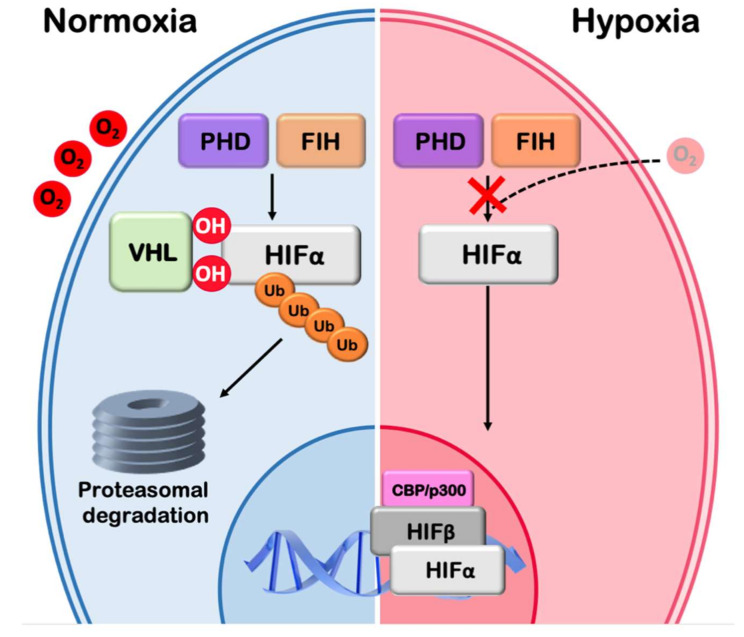
The hypoxic signaling pathway. The hypoxic signaling pathway: Under normoxia HIFα is hydroxylated by PHD and FIH hydroxylases in an oxygen dependent manner. Hydroxylation by PHD leads to pVHL and E3 ubiquitin ligase recruitment, ubiquitination of HIFα and subsequent proteasomal degradation. Hydroxylation by FIH leads to transcriptional inactivation. Low O_2_ inactivates both PHD and FIH hydroxylases, HIFα is stabilized, enters the nucleus, dimerizes with HIF-1β (ARNT), binds to specific DNA elements (hypoxia responsive elements) and, in association with transcription coactivators such as CBP/p300, stimulates the transcription of hypoxia target genes.

Multicellular organisms have evolved homeostatic mechanisms in order to maintain cellular and tissue function upon changes of oxygen tension. Exposure of human tissues or cells to reduced oxygen concentration (a condition known as hypoxia) is encountered both during physiological (e.g., high altitude, intense exercise and embryogenesis) and pathological processes (e.g., ischemia and cancer) [71,72,73]. Hypoxia promotes dramatic reprogramming of gene expression followed by a cascade of events including switching to anaerobic production of energy, modulation of lipid metabolism, increased transport and delivery of oxygen, invasion and metastasis (in the case of cancer cells), which, overall, facilitate adaptation and survival of cells in the hypoxic environment [74,75,76]. Essential to these responses are the hypoxia-inducible transcription factors HIF-1 and HIF-2 (EPAS1), which are also implicated in tumor progression, invasion, metastasis and resistance to radiotherapy [77,78,79]. Under normal oxygen conditions the regulatory alpha subunit (HIFα) of HIFs is continuously produced and destroyed, in a process involving modification by prolyl hydroxylases (PHDs), pVHL-mediated polyubiquitination and subsequent proteasomal degradation (Figure 2). In addition, the presence of oxygen reduces HIF transcriptional activity by promoting the activity of the factor inhibiting HIF (FIH), an asparaginyl hydroxylase, which hydroxylates an asparagine residue in the C-terminal transactivation domain of HIFα and prevents the recruitment of HIF coactivators CBP/p300 [80,81]. However, when oxygen concentration is low, hydroxylation by PHDs and FIH is impaired, HIFα is stabilized, translocates to the nucleus and dimerizes with ARNT (HIF-1β) to form HIF dimers that bind to hypoxia-responsive elements (HREs) in the promoters/enhancers of their target genes [82,83].

## 6. The Interplay between Sumoylation and the Hypoxic Response

A number of findings support that covalent attachment of proteins to SUMO is required for the adequate activation of the response to hypoxia and the ensuing signaling cascade. This may concern modification of particular proteins of the hypoxic signaling cascade or alterations of global sumoylation levels in hypoxic cells triggered by changes in the SUMO conjugation machinery [59,84] In this review, we will discuss both cases and their relevant effects on the response to low oxygen concentrations.

### 6.1. Changes in Global Sumoylation under Ischemia

At the tissue level, hypoxia is often manifested as part of ischemia (restriction of blood flow), which is characterized by both a severe lack of oxygen and deprivation of nutrients and can lead to cell death in the infarct areas. Significant changes in global sumoylation and SUMO machinery components caused by ischemia have been reported in both heart and brain of animals. A set of studies has demonstrated that SUMO-conjugation is globally increased after ischemia as a stress protective mechanism [85,86,87]. Specifically, a massive increase of SUMO was observed in the brain (hippocampus and striatum) of rats with middle cerebral ischemia [88,89,90]. In a transgenic mouse model in which Ubc9 was constitutively overexpressed, leading as expected to an increase of SUMO1- and SUMO2/3-conjugated proteins, cerebral infarct volumes were decreased compared to wild-type mice [91]. In line with these results, overexpression of SUMO1 or SUMO2 in cortical neurons and SHSY5Y cells increased survival after oxygen and glucose deprivation (known as the OGD model that mimics ischemic conditions in cells) and, in agreement, depletion of SUMO1 reduced cell survival after OGD [92]. However, there have also been studies showing that SUMO2/3 conjugation was not changed or even reduced in cultured cells under OGD conditions when OGD was preceded by short OGD or hypothermic preconditioning, in a model of delayed ischemic tolerance [93]. It may, therefore, be possible that modulation of sumoylation of proteins under ischemia is mostly a result of cell damage rather that an adaptive mechanism to the ischemic stress.

Concerning the SUMO machinery components, higher SENP1 levels were reported in cultured neurons during OGD [94,95], and in human and mouse myocardium after ischemia/reperfusion [94,95], and SENP3 was shown to exhibit a protective role against myocardial ischemia/reperfusion injury [96]. These studies have not led to the identification of specific proteins that are regulated by sumoylation under ischemia or proteins that are responsible for the protective effect of sumoylation.

Using a SILAC-based proteomic approach, Yang and coworkers [97] have identified several SUMO2/3 targets (mostly nuclear proteins involved in gene expression) with significantly altered sumoylation levels in neuroblastoma cells under OGD conditions. These included two PIAS SUMO ligases, which may, therefore, also play a role in the global increase of SUMO conjugation under ischemia. The same study also revealed that the activation of SUMO2/3 is required for stimulation of ubiquitin conjugation in OGD-stressed cells, indicating a previously unanticipated interplay between the ubiquitin and SUMO conjugation machineries and their cooperation in protection from ischemic stress. In another study, sumoylation of the brain sodium/calcium exchangers NCX3 was shown to be required for the protective effect of SUMO1 during ischemic preconditioning [98].

### 6.2. Changes in Global Sumoylation under Hypoxia

Although changes in the sumoylation pattern have been observed widely in ischemic animals or brain tissue and OGD models, there are few reports on the modulation of sumoylation under hypoxia alone (i.e., without concomitant nutrient starvation). It should be stressed here that normal oxygen tension in most healthy tissues corresponds to 4–6% oxygen (compared to 21% oxygen in inhaled air), while in hypoxic tissues (such as solid tumors) it can range between <0.5%, considered as severe hypoxia, and 1–3%, considered as mild hypoxia and often used as hypoxic conditions in cell cultures [99].

Increased global SUMO-conjugation upon hypoxia was initially reported as a result of increased SUMO1 expression in T84 colon cells cultured under hypoxia and in the brain and heart of mice exposed to 10% oxygen [100,101]. Others and we have investigated the SUMO proteome of cells that have been kept for 24–48 h under the hypoxic condition (1% oxygen) [102,103]. By using a SILAC-quantitative proteomic approach, we did not observe a massive increase in the number of SUMO1 or SUMO2/3 modified proteins but rather significant sumoylation-status changes of a small group of proteins [103]. These proteins were mostly transcription factors (like TFAP2a, a protein that could be involved in the hypoxic response [103]). Kunz et al. [102] also identified alteration in sumoylation in a subset of proteins, such as the SUMO machinery enzymes RanBP2 and PIAS2 E3 SUMO ligases and regulators of transcription, by analyzing normoxic and hypoxic cells using comparative mass spectrometry. A recently observed downregulation of Ubc9 acetylation via SIRT1 under hypoxia could be responsible for the sumoylation alterations of these specific targets [104]. Specifically, it was shown that acetylation of Ubc9 could downregulate sumoylation of a subset of proteins that contain a negatively charged amino acid-dependent sumoylation motif (ψKXE/D classical motif followed downstream by acidic residue cluster) and not proteins with classic motif or SIM. Moreover, Kunz et al. [102] observed inactivation of SENP1 and SENP3 under hypoxia without changes in their expression levels. It is proposed that inactivation of these SENPs could be linked to the modulation of sumoylation of specific protein targets or to changes in the SUMO2/3 maturation process, thus influencing SUMO2/3 availability [102].

From all the above it can be assumed that distinct mechanisms of sumoylation characterize responses to hypoxic or ischemic stimuli. Even though, an induction of SUMO2/3 conjugation is considered crucial for the survival of cells under ischemic (and often lethal) stress, modulation of the sumoylation of a subset of proteins is more profound under hypoxia and could serve as part of the cellular adaptation and physiological response to low oxygen, a condition largely anticipated by the cellular machinery.

## 7. Impact of Sumoylation on Key Players of the Response to Hypoxia

**Figure 3:**
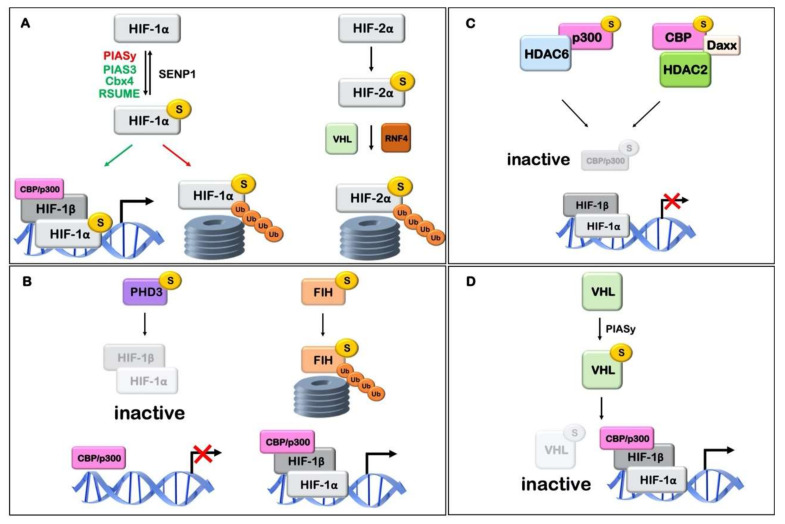
Schematic representation of the association of hypoxic signaling agents with SUMO and its effect on hypoxia inducible factor (HIF) stability and activity. (**A**) HIFα sumoylation affects its transcriptional activity. E3 ligases PIAS3, Cbx4 and RSUME may mediate sumoylation and activation of HIF-1, while HIF-1α sumoylation by E3 ligase PIASy may lead to its proteasomal degradation. Desumoylation of HIF-1α by sumo isopeptidase SENP1 may lead to stabilization. HIF-2α sumo modification leads to VHL and RNF4 dependent ubiquitination and proteasomal degradation. (**B**) Sumoylation of HIFα hydroxylases affects their activity. Modification of PHD3 causes inhibition of HIF-1 transcriptional activity, without, however, affecting HIF-1α protein stability. Sumoylation of FIH promotes its proteasomal degradation, thus enhancing the transcriptional activity of HIF-1α. (**C**) Sumoylation of p300 and CBP affects HIF-1 activity. SUMO conjugation of p300 or CBP leads to transcriptional repression of HIF-1 target genes mediated by SUMO-dependent recruitment of HDAC6 and HDAC2/DAXX, respectively. (**D**) Sumoylation of pVHL by PIASy promotes its deactivation leading to HIF stabilization and activation. Relevant references and details can be found in Table 1.

SUMO can directly interact and modify a set of proteins that participate in the hypoxic signaling cascade and this modification is critical for their function. The influence of sumoylation on key elements of the hypoxic pathway is discussed below and is schematically described in Figure 3 and Table 1.

### 7.1. HIFα

The most important players of the hypoxic response pathway are the HIF transcription factors. In addition to hydroxylation (see above) the oxygen sensitive HIFα subunits are also regulated by additional post-translational modifications such as acetylation, phosphorylation, *S*-nitrosylation, ubiquitination and sumoylation. These modifications may influence not only their protein stability but also their transcriptional activity [84,120].

HIF-1α has been long known to be sumoylated at residues K391 and K477 [101,105,111] but the effect of these modifications is still unclear. According to one scenario, sumoylation of HIF-1α increases both its stability and transcriptional activity [108,111]. Overexpression of SUMO1 in 293T cells resulted in HIF-1α stabilization and subsequent increase of its transcriptional activity [111]. In agreement, the RWD-containing sumoylation enhancer (RSUME) was shown to be induced by hypoxia and enhance HIF-1α sumoylation, which promoted HIF-1α stabilization and transcriptional activity [108,109]. However, in an alternative contrary scenario, sumoylation of HIF-1α stimulates its VHL-mediated ubiquitination and inhibits its activity [105]. Berta et al. [105] found that HIF-1α could be modified by SUMO1 and SUMO2/3 in vitro and this modification was facilitated by SUMO E3 ligase RanBP2/Nup538. By using sumoylation deficient HIF-1α forms, they further showed that a lack of SUMO modification increased HIF-1α transcriptional activity [105]. In support, Cheng et al. [107], by performing experiments in *SENP1*−/− mice, demonstrated that when SENP1 was deleted, sumoylation of HIF-1α was increased and led to its degradation in a VHL- and ubiquitin/proteasome-dependent manner, while expression of SENP1 was essential for HIF-1 transcriptional activity. More recent studies [121,122] are in agreement with these results, reporting that SENP1 increased the stabilization and transcriptional activity of HIF-1α in hypoxia via desumoylation in hepatocellular carcinoma (HCC) and ovarian cancer cells. High SENP1 expression levels were also associated with upregulation of glycolytic enzymes, which are known HIF-1 targets, in clear cell renal cell carcinoma [123]. In addition, it was shown that SENP1 is a direct target of HIF-1 suggesting the operation of a positive feedback loop that enhances HIF-1 activity via SENP1 expression [121,124]. The cause of this apparent controversy between the suggested roles of HIF-1α sumoylation may be the fact that when the equilibrium in the expression of SUMO machinery components like SUMO or SENP is disturbed (as in silencing or overexpression experiments), not only HIF-1α but also several other elements of the hypoxic pathway are simultaneously affected (see below). This can result in variable outcomes (in terms of HIF-1 activity) that depend on the cellular context.

There is also controversy regarding the different E3 ligases that interact with HIF-1α and their effects. PIASy was the first SUMO E3 ligase reported to interact with HIF-1α. PIASy negatively regulated HIF-1α stability and activity by enhancing sumoylation of hypoxia-induced HIF-1α [106]. Other studies [110] suggest that Cbx4 (a member of the polycomb group and a previously characterized SUMO E3 ligase) is responsible for the sumoylation of HIF-1α under hypoxia and in contrast to PIASy, Cbx4 was shown to increase HIF-1 activity and, subsequently, hypoxia-induced VEGF expression and angiogenesis in HCC cells and transplanted mice HCC models. A third SUMO E3 ligase that was recently found to interact with HIF-1α was PIAS3 [125]. PIAS3 enhanced the stabilization and transcriptional activity of HIF-1α, but this effect was independent of its SUMO E3 ligase activity, suggesting that it did not involve HIF-1α sumoylation. In conclusion, HIF-1α appears to interact with various components of the SUMO modification machinery, with different outcomes on HIF-1 transcriptional activity, depending on the cell type and experimental approaches. 

Sumoylation of HIF-2α, on the other hand, is not so extensively studied. HIF-2α can be sumoylated in vitro [126] and its sumoylation at K394 in HeLa cells leads to VHL-and RNF4 (a SUMO-targeted ubiquitin E3 ligase)-dependent ubiquitination and proteasomal degradation [112]. 

### 7.2. HIF-1β (ARNT)

In addition to the oxygen-dependent subunits of the HIF heterodimer, its constitutively expressed subunit HIF-1β or ARNT is also subject to post-translational regulation. Endogenous and overexpressed ARNT was shown to be modified in vivo under normoxia (in HeLa and MCF7 cells) and in vitro (using a recombinant GST-ARNT fragment) by SUMO1 at K245 within its PAS domain, a region required for forming complexes with members of the bHLH/PAS protein family including HIF-α as well as the aryl hydrocarbon receptor (AhR). Sumoylation of ARNT by the PIAS1 E3 ligase inhibited its transcriptional capacity and also its ability to interact with PML in PML bodies [113]. However, it is not clear whether this has any significant effect on the activity of HIFs.

### 7.3. PHD and FIH Hydroxylases

The prolyl-4-hydroxylase domain (PHD) proteins that modify HIFα are essential to the maintenance of oxygen homeostasis and are tightly regulated. PHD3 (also known as EGLN3) participates mainly in a negative-feedback loop in response to prolonged hypoxia [114]. PHD3 was found sumoylated when ectopically coexpressed with SUMO2/3 [115] within a cluster of four possible lysine residues (K222, K223, K224 and K231) at non-SUMO consensus sites. Moreover a SUMO conjugated-PHD3 form was a more potent inhibitor of HIF-1 transcriptional activity under hypoxia than wild type PHD3, without, however, affecting HIF-1α protein stability [115].

More recently, a study has shown that FIH, the asparaginyl hydroxylase that negatively regulates the transcriptional activity of HIF-1α, is also a target of SUMO2/3 in low oxygen conditions (3% O_2_) in JEG-3 human choriocarcinoma cells. Sumoylation of FIH in hypoxia promotes its proteasomal degradation, thus enhancing the transcriptional activity of HIF-1α [116].

### 7.4. CBP/p300

p300 and CREB binding protein (CBP) are homologous transcriptional coactivators that are utilized by many DNA binding proteins to facilitate transcriptional activation [127]. CBP/p300 is involved in the HIF-mediated transcriptional activation of hypoxia target genes and physically interact with the C-TAD of HIFα [128,129]. p300 is covalently modified by SUMO1 at K1020 and K1024 while CBP at K999, K1034 and K1057 [117,118,130]. p300 sumoylation lead to transcriptional repression mediated by SUMO-dependent recruitment of HDAC6 to p300. Desumoylation of p300 by SENP3 has been shown to enhance HIF-1 transcriptional activity, verifying the previous observation [70].

The BRD, PHD and ZZ domains of CBP interact with SUMO1 and Ubc9 and mediate sumoylation of the cell cycle regulatory domain 1 (CRD1) of CBP [131]. As is the case for p300, modification by SUMO may negatively modulate the transcriptional activity of CBP by recruiting Daxx, a known transcriptional repressor, which facilitates the association of histone deacetylase HDAC2 with CBP and subsequent transcriptional repression [118]. 

### 7.5. von Hippel-Lindau Protein (pVHL)

Under normal oxygen conditions, hydroxylated HIF-1α is rapidly targeted for proteasome-mediated degradation through the elongin BC/Cul2/VHL E3 ubiquitin ligase complex, which requires recognition of the hydroxylated sites by the von Hippel Lindau tumor suppressor protein (pVHL) [132]. Upregulation of the SUMO E3 ligase PIASy by hypoxia leads to its interaction with pVHL and to pVHL sumoylation on K171 [119]. This modification inhibits the tumor suppressor activity of pVHL and the pVHL-dependent degradation of HIFα, leading to HIF activation. However, this is in contrast to the aforementioned inactivation of HIF-1α upon its PIASy mediated sumoylation [106]. One study on pVHL and RSUME interaction, suggests that RSUME can interact with VHL independently of HIF-1α. RSUME promotes sumoylation of pVHL, which leads to pVHL inactivation, and subsequent stabilization of HIF-α under normoxic conditions [133].

## 8. Sumoylation of Targets That Indirectly Affect the Hypoxic Pathway

Besides the impact of sumoylation on the immediate regulators of the hypoxic pathway (shown in Figure 3 and Table 1), sumoylation is known to influence the function of many other proteins that are indirectly related to the hypoxic signaling pathway. For example, the Ras/MAPK and PI3K/Akt kinase pathways that both influence HIFα and its transcriptional activity [134,135,136,137,138] are modulated by sumoylation. Specifically, the MEK1 and MEK2 kinases are sumoylated and this leads to the inhibition of their kinase activity [139]. On the other hand, sumoylation of Akt enhances its kinase activity [140] (for review see [141]).

The NF-kB pathway is known to be activated under hypoxia [142] and can lead to transcriptional induction of HIF-1α in immune system cells [143]. Sumoylation of IkBa by SUMO1 or SUMO2/3 was reported to differentially regulate the NF-kB signaling pathway [144,145]. Moreover, ectopic expression of SUMO-2 has been shown to block nuclear translocation of the p65 subunit of NF-kB [146]. Very recently, overexpression of SENP1 in microglia was shown to decrease sumoylation of NEMO and inhibited NF-kB activation and the inflammatory response upon intermittent hypoxia [147].

Finally, sumoylation may also influence downstream targets of the HIF axis like the VEGF-dependent responses. The VEGF-induced angiogenic response was inhibited by SENP1 silencing, mainly because of VEGFR2 sumoylation and its subsequent accumulation at the Golgi apparatus [148]. Interestingly, products of HIF-1 gene targets, such as glucose transporters GLUT4 and GLUT1, also interact with Ubc9 and can be conjugated to SUMO [149,150]. All the above show that sumoylation can interfere by multiple ways (directly and indirectly) in the hypoxic signaling response.

## 9. Conclusions and Future Perspectives

Sumoylation of proteins contribute to nearly all aspects of cell biology. Most importantly, all the aforementioned data support the notion that sumoylation/desumoylation represents an additional control point in the cell response to low oxygen concentration. Even though significant work has been made towards elucidating the role of SUMO modification under low oxygen conditions, especially in ischemia, a lot of controversy and many unanswered questions still remain. For example, is it global or targeted sumoylation that influences mostly the cell response to ischemia/hypoxia? Is the involvement of SUMO modification in the hypoxic response a general or a tissue/cell specific phenomenon? In other words, are sumoylation/desumoylation reactions essential parts of the response to hypoxia or do they fine-tune the response taking into account the cellular context and integrating other environmental cues? Given the variable effects of sumoylation on the different components of the hypoxia response pathway, which one is the SUMO-affected step that is most important for the end result, i.e., better adaptation to low oxygen conditions and/or tumor growth? How does the intracellular location of the SUMO conjugation enzymes influence their access to sumoylation targets, and can this explain the apparent inconsistencies? Additionally, finally, how does the SUMO machinery sense oxygen levels? Certain possibilities such as hypoxia-induced changes on the sumoylation of E3 SUMO ligases or the deacetylation of the UBC9 enzyme or the activity of specific SENPs have been already discussed [102,104]. However, it is still unclear how these changes can be elicited by low oxygen concentration. One possibility entails the transcriptional regulation of SUMO machinery components, directly or indirectly, by HIFs activated by hypoxia. On the other hand, more rapid and local non-transcriptional events that are also known to be triggered by hypoxia, such as activation of specific kinases [151] or changes in redox equilibrium [152], could also influence sumoylation. A thorough characterization of these pathways is necessary for providing answers in the near future.

As hypoxia is a hallmark of serious pathological conditions such as cancer and ischemia, an important challenge for the future is to provide better understanding of the connections between sumoylation and the various steps along the hypoxia response pathway so as to control them and open the way to novel molecular interventions. As new emerging molecules targeting sumoylation show promise in the treatment of hematological malignancies [153], they may also be suitable for modulating the SUMO machinery and exhibiting therapeutic potential in cases of solid hypoxic tumors.

## Figures and Tables

**Table 1:** The effect of sumoylation on key elements of the hypoxic pathway.

Protein	Effect of SUMOylation	Details	Reference
HIF-1α	n.d.	SUMO1 is increased under hypoxia and directly interacts with HIF-1α	[101]
	Negative: ↑ degradation and/or ↓ activity	HIF-1α modification by RanBP2 inhibits HIF-1 activity	[105]
		PIASy-dependent modification and destabilization of HIF-1α is reversed by SENP1 (a HIF-1 target) under hypoxia	[106,107]
	Positive: ↑ protein stability and/or ↑ activity	Overexpression of SUMO1 or RSUME or Cbx4 stabilizes and/or activates HIF-1α.	[108,109,110,111]
HIF-2α	Negative: ↑ degradation	Sumoylation leads to VHL/RNF4-dependent ubiquitination and degradation of HIF-2α	[112]
ARNT	Negative: ↓ activity	Sumoylation under normoxia inhibits transcriptional capacity of ARNT and its interaction with PML	[113]
PHD3	↓ HIF-1 activity	PHD3 sumoylation represses HIF-1 transcriptional activity under hypoxia, without affecting its stability or PHD activity	[114,115]
FIH	↑ FIH degradation ↑ HIF-1 activity	FIH sumoylation under hypoxia promotes its degradation and enhances HIF-1α transcriptional activity	[116]
CBP/p300	↓ coactivator activity	Sumoylation of CBP or p300 recruits transcriptional repressors (Daxx/HDAC2 or HDAC6, respectively)	[117,118]
	↓ HIF-1 activity	De-sumoylation of p300 by SENP3 enhances HIF-1 activity	[70]
pVHL	↓ VHL interaction with HIFα ↑ HIF activity	pVHL sumoylation by PIASy inhibits its interaction with HIFα	[119]
